# Comment on “Clonal hematopoiesis of indeterminate potential and breast cancer risk”

**DOI:** 10.1097/JS9.0000000000003339

**Published:** 2025-09-08

**Authors:** Jing Feng, Zhen Zhang, Ye Zhang

**Affiliations:** a Department of Breast Center, Mianyang Central Hospital, School of Medicine, University of Electronic Science and Technology of China, Mianyang, Sichuan, People’s Republic of China; b Department of Neurosurgery, Mianyang Central Hospital, School of Medicine, University of Electronic Science and Technology of China, Mianyang, Sichuan, People’s Republic of China

*Dear Editor,*

We read with great interest the cohort study by Lai *et al*^[1]^, reporting a 19% higher risk of breast cancer among individuals with clonal hematopoiesis of indeterminate potential (CHIP) in the UK Biobank. This finding has important implications for risk stratification and early detection. The use of high-depth sequencing and rigorous adjustment for confounders provides a strong framework for evaluating somatic mutations as cancer risk factors. We commend the authors and offer several suggestions to enhance the scientific and translational impact of their work. No generative artificial intelligence tools were used in the design, execution, analysis, or writing of this correspondence, and our article is compliant with the TITAN Guidelines 2025^[2]^.

First, the UK Biobank’s volunteer-based recruitment may underrepresent socioeconomically disadvantaged and medically underserved groups, potentially underestimating CHIP prevalence and effect size. Validation in external, demographically diverse cohorts – such as The Cancer Genome Atlas (TCGA) or other population-based biobanks – would strengthen generalizability across ethnic and clinical backgrounds^[3,4]^. Meta-analytic integration of multiple datasets could also improve power for less common CHIP driver mutations.

Second, although individuals with diagnosed breast cancer were excluded, undetected preclinical cases may still bias results. Landmark analyses excluding cases in the first 1–2 years or modeling CHIP status as a time-varying covariate would better address temporality and reduce reverse causation^[5]^.

Third, while ATM and DNMT3A mutations were significant drivers, other common genes (e.g., TET2, ASXL1) were not. Stratification by variant allele frequency, mutation class, and co-mutation patterns may clarify gene-specific risk profiles. Adjustment for germline polygenic risk scores would help determine whether CHIP effects are independent of inherited predisposition.

Fourth, the proposed inflammatory mediation lacks direct biomarker evidence. Longitudinal C-reactive protein and interleukin-1β measurements, combined with functional studies of CHIP-driven immune phenotypes, could clarify mechanisms linking clonal expansion to breast epithelial transformation.

Finally, the clinical translation of these findings warrants discussion. Should somatic mutation profiling be incorporated into screening for postmenopausal women? What are the cost-effectiveness and ethical considerations of integrating CHIP into risk prediction models? Addressing these questions is key for moving from molecular association to preventive application.

In summary, this study provides important evidence linking CHIP to breast cancer susceptibility. We encourage external validation, temporality-sensitive modeling, gene-level analysis, and biomarker-based mechanistic studies to support clinical implementation.

## Added value

This correspondence identifies actionable steps to advance CHIP–breast cancer research: external validation in diverse populations, temporality-sensitive analysis (landmark and time-varying exposure models), gene-level risk profiling (variant allele frequency, mutation class, co-mutations, polygenic risk adjustment), and biomarker-based assessment of inflammatory mediation to support risk-adapted prevention strategies.

## Learning points

- Replicate findings in demographically diverse cohorts; use meta-analysis to evaluate rare CHIP drivers.
- Apply landmark and time-varying exposure designs to minimize reverse causation.
- Incorporate longitudinal inflammatory biomarkers and functional immunophenotyping to test mechanistic mediation and inform integration into risk models and screening strategies.

## Data Availability

No new data were generated or analyzed in this correspondence. All information discussed is based on previously published sources, which are properly cited within the article.

## Research registration unique identifying number (UIN)

Not applicable. This letter does not report original clinical research requiring registration.
